# Impact of geriatric nutritional risk index on outcomes after gastrectomy in elderly patients with gastric cancer: a retrospective multicenter study in Japan

**DOI:** 10.1186/s12885-022-09638-6

**Published:** 2022-05-12

**Authors:** Tomoyuki Matsunaga, Hiroaki Saito, Tomohiro Osaki, Sadamu Takahashi, Akemi Iwamoto, Kenji Fukuda, Kenjiro Taniguchi, Hirohiko Kuroda, Tsutomu Takeuchi, Kenji Sugamura, Kenichi Sumi, Kuniyuki Katano, Yuji Shishido, Kozo Miyatani, Yoshiyuki Fujiwara

**Affiliations:** 1 Division of Gastrointestinal and Pediatric Surgery, Department of Surgery, School of Medicine, Tottori University Faculty of Medicine, 36-1 Nishi-cho, Yonago, 683-8504 Japan; 2 Department of Surgery, Japanese Red Cross Tottori Hospital, Tottori, 680-8517 Japan; 3 Department of Surgery, Tottori Prefectural Central Hospital, Tottori, 680-0901 Japan; 4 National Hospital Organization, Hamada Medical Center, Hamada, 697-8511 Japan; 5 Divisions of Digestive Surgery, Tottori Prefectural Kousei Hospital, Kurayoshi, 682-0804 Japan; 6 Department of Surgery, Sanin Rosai Hospital, Yonago, 683-8605 Japan; 7 Department of Surgery, Yonago Medical Center of National Hospital Organization, Yonago, 683-0006 Japan; 8 Department of Surgery, Japanese Red Cross Masuda Hospital, Masuda, 698-8501 Japan; 9 Department of Surgery, Tottori Seikyo Hospital, Tottori, 680-0833 Japan; 10 Department of Surgery, Yasugi Municipal Hospital, Yasugi, 692-0404 Japan; 11 Department of Surgery, Hakuai Hospital, Yonago, 683-0853 Japan; 12 Department of Surgery, The Nanbu Town National Health Insurance Saihaku Hospital, Nanbu, 683-0323 Japan

**Keywords:** Elderly patient, Gastric cancer, Prognosis, Geriatric nutritional risk index

## Abstract

**Background:**

Several studies investigated the utility of inflammation and nutritional markers in predicting the prognosis in patients with gastric cancer; however, the markers with the best predictive ability remain unclear. This retrospective study aimed to determine inflammation and nutritional markers that predicted prognosis in elderly patients over 75 years of age undergoing curative gastrectomy for gastric cancer.

**Methods:**

Between January 2005 and December 2015, 497 consecutive elderly gastric cancer patients aged over 75 years underwent curative gastrectomy in 12 institutions. The geriatric nutritional risk index (GNRI), prognostic nutritional index, neutrophil/lymphocyte ratio, platelet/lymphocyte ratio, and C-reactive protein/albumin ratio were examined as prognostic markers for overall survival (OS) and disease-specific survival (DSS) using area under the curve (AUC) using receiver operating characteristic (ROC) curve analysis.

**Results:**

The GNRI had the highest AUC and predictive value for both OS (0.637, *p* < 0.001) and DSS (AUC 0.645, *p* < 0.001). The study cohort was categorized into the high and low GNRI groups based on the optimal GNRI cut-off values for OS (97.0) and DSS (95.8) determined with the ROC analysis. For both OS and DSS, there was a significant correlation between the GNRI and several clinicopathological factors including age, body mass index, albumin, American Society of Anesthesiologists physical status score, depth of tumor invasion, lymph node metastasis, lymphatic invasion, pathological stage, operation duration, bleeding, procedure, approach, death due to primary disease, and death due to other disease. The GNRI remained a crucial independent prognostic factor for both OS (Hazard ratio [HR] = 1.905, *p* < 0.001) and DSS in multivariate analysis (HR = 1.780, *p* = 0.043).

**Conclusions:**

Among a panel of inflammation and nutritional markers, the GNRI exhibited the best performance as a prognostic factor after curative gastrectomy in elderly patients with gastric cancer, indicating its utility as a simple and promising index for predicting OS and DSS in these patients.

**Supplementary Information:**

The online version contains supplementary material available at 10.1186/s12885-022-09638-6.

## Background

Aging is an inevitable process for all humans, and the global aging population is increasing in parallel to improved quality of life and medical advances [1]. Elderly individuals constitute one of the most vulnerable populations and are at high risk for various nutritional issues and comorbidities including gastric cancer [2–4]. Indeed, the rate of elderly patients with gastric cancer is increasing and their high mortality rate is an issue [5, 6]. Therefore, prognostic prediction is important in elderly patients with gastric cancer. Importantly, staging, which is generally used to predict cancer prognosis [7], is not sufficient in elderly patients with gastric cancer [6, 8].

In recent years, various inflammation markers and nutritional indicators, such as the neutrophil/lymphocyte ratio (NLR) and platelet/lymphocyte ratio (PLR), prognostic nutritional index (PNI), and C-reactive protein (CRP)/albumin ratio (CAR), have been demonstrated to exhibit high utility in predicting surgical complications and prognosis in various cancers [9–11]. Although several studies have investigated the utility of these markers in patients with gastric cancer [12–16], the identification of those markers having the best predictive ability in elderly patients with gastric cancer remains unclear. In particular, no studies to date have determined the most useful factors for prognosis in patients over 75 years of age undergoing curative gastrectomy for gastric cancer. The recently developed geriatric nutritional risk index (GNRI) has been shown to exhibit utility as a prognostic factor in various carcinomas [17–19]. Importantly, the GNRI can be easily calculated from routine hematological data including serum albumin, height, and weight. These readily available parameters can reflect the survival risk in various cancers and have demonstrated utility in elderly patients [17, 20].

In the present retrospective multicenter study, we aimed to determine inflammation markers and nutritional indicators with the best prognostic utility for outcomes after gastrectomy in elderly patients over 75 years of age with gastric cancer.

## Methods

### Patients

**Fig. 1:**
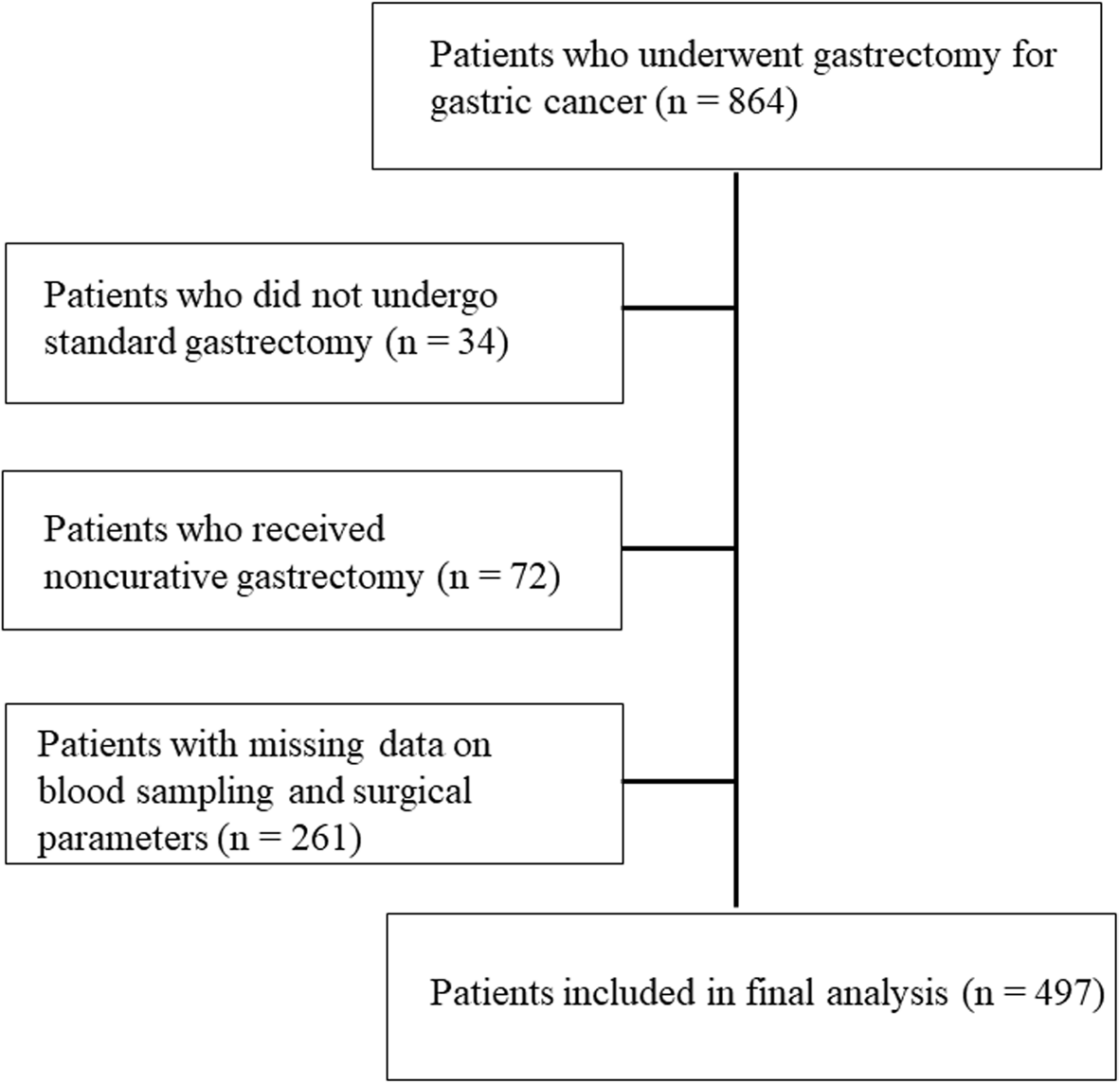
CONSORT diagram

From January 1, 2005 to December 31, 2015, 864 consecutive patients aged 75 years or older who were diagnosed with gastric cancer underwent gastrectomy in 14 institutions participating in the present study. Among these, 34 patients who underwent procedures other than standard gastrectomy such as local resection, 72 patients who underwent non-curative gastrectomy, and 261 patients with missing data on blood sampling results or surgical factors were excluded (Fig. 1). A total of 497 patients were included in the final analysis. The clinicopathological findings were determined according to the Japanese gastric cancer treatment guidelines [21]. Clinical data, including age, sex, American Society of Anesthesiologists physical status (ASA-PS) score, histology, depth of tumor invasion, lymph node metastasis, pathological stage, operation duration, bleeding, procedure, approach, and adjuvant chemotherapy, were collected from the databases of the participating institutions. Included comorbidities were cardiac and respiratory diseases and diabetes mellitus. In this study, cardiac disease was defined as the diagnosis of New York Heart Association class III or IV cardiac disease or arrhythmia requiring mechanical support, pulmonary disease was defined as % vital capacity of less than 60% or percent predicted forced expiratory volume in 1 s of less than 50%, and diabetes mellitus was defined as the use of oral hypoglycemic drugs or insulin with a higher hemoglobin A1c level above the reference value for each study institution. Patients were periodically checked for cancer recurrence via physical examination and blood tests performed every 3 months following hospital discharge. Computed tomography (CT) was performed at least every 6 months after surgery. Data on recurrence patterns and causes of death were extracted from the clinical records and positron emission tomography/CT results. In patients with difficulty in obtaining follow-up data, direct inquiries were made with their families.

### Inflammation and nutritional factors

Peripheral counts of neutrophils, lymphocytes, and platelets and serum levels of albumin, carcinoembryonic antigen, and carbohydrate antigen 19–9 were collected from the medical records. Preoperative blood tests were performed within 7 days before surgery. The GNRI was calculated using the following formula: GNRI = 14.89 × serum albumin level (g/dL) + 41.7 × [current body weight (kg)/ideal body weight (kg)], where ideal weight was defined as 22 × [height (m)]^2^ [22]. In patients with greater than the ideal body weight, the ratio of the actual body weight to the ideal body weight was set to 1 [22]. The NLR and PLR were calculated by dividing the peripheral neutrophil and platelet counts by the peripheral lymphocyte count, respectively [12, 23]. The PNI was calculated using the following formula: PNI = 10 × serum albumin level + 0.005 × total lymphocyte count [24]. The CRP/albumin ratio was calculated by dividing the serum CRP level by the serum albumin level [12]. The following modified Glasgow prognostic scoring system, which combines CRP and serum albumin levels, was used: score of 0, normal albumin (≥3.5 g/L) and normal CRP (≤10 mg/L); score of 1, elevated CRP (> 10 mg/L) and low albumin (< 3.5 g/L); and score of 2, elevated CRP level (> 10 mg/L) and low albumin (< 3.5 g/L) [25].

### Statistical analysis

Continuous variables were expressed as means ± standard deviation. The χ^2^ or Fisher’s exact test was used to compare categorical variables, and Student’s *t*-test was used to compare continuous variables. Survival curves were calculated using the Kaplan–Meier method, and differences between survival curves were examined using the log-rank test. The Cox proportional hazards model was used to perform univariate and multivariate analyses of prognostic factors for overall survival (OS) and disease-specific survival (DSS). Receiver operating characteristic (ROC) analysis for OS was performed to determine the cutoff value of age, operation duration, and bleeding amount. A *p* value of < 0.05 was considered to indicate statistical significance. SPSS for Windows version 24 (IBM, Armonk, NY, USA) was used for all statistical analyses.

## Results

### The utility of GNRI according to OS

**Fig. 2:**
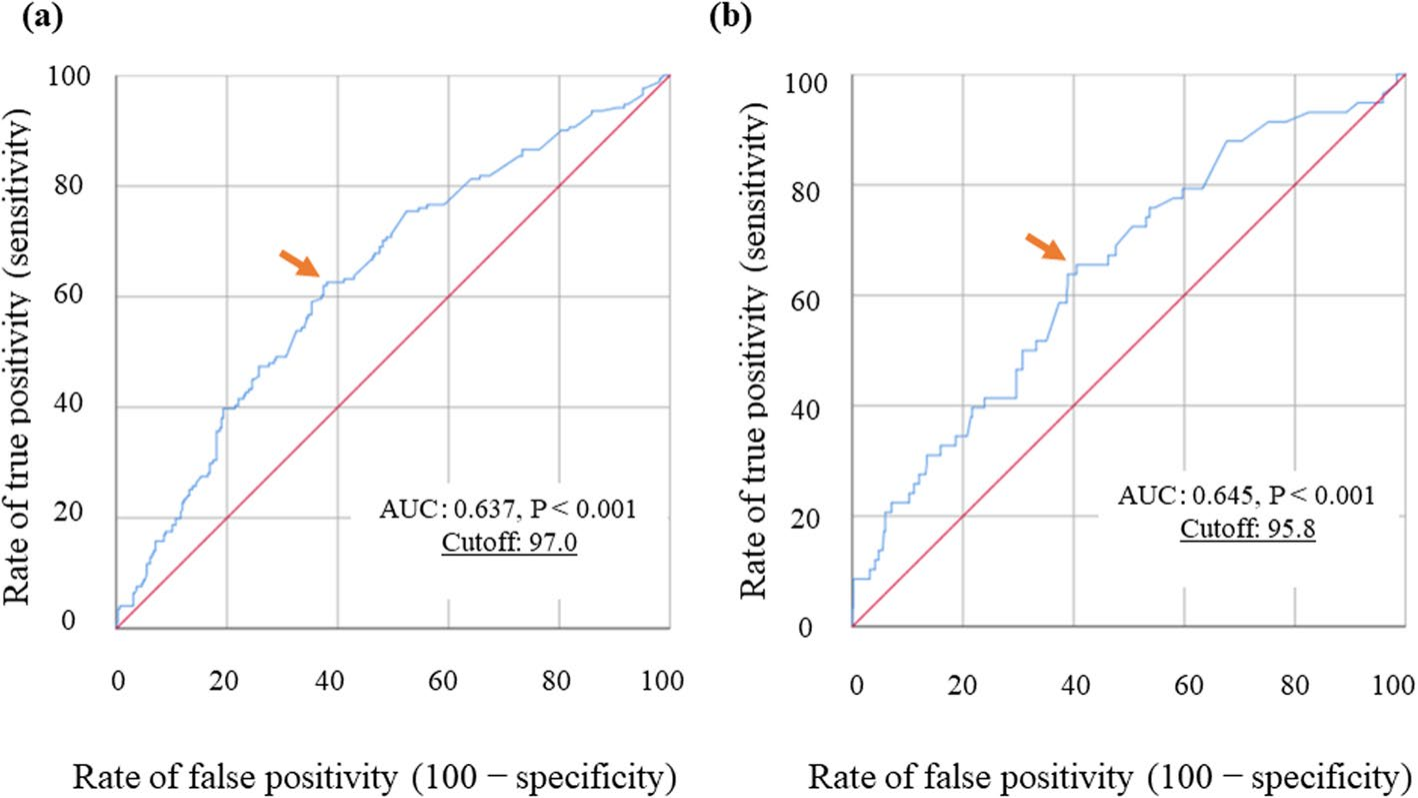
ROC curves of GNRI for OS (**a**) and DSS (**b**). Arrows indicate optimal cutoff values. Abbreviations: AUC, area under the receiver operating characteristic curve; DSS, disease-specific survival; GNRI, geriatric nutritional risk index; OS, overall survival; ROC, receiver operating characteristic

Table 1 shows the area under the curve (AUC) for each potential prognostic factor, which was determined using ROC curve analysis for OS. Among these, the GNRI had the highest AUC and the highest predictive value. Using the optimal GNRI cutoff value of 97.0 for OS determined with the ROC analysis (Fig. 2a), the study patients were divided into the high GNRI (GNRI^High^, *n* = 269) and low GNRI (GNRI^Low^; *n* = 228) groups. Table 2 summarizes the relationship between the GNRI and characteristics of the study patients based on OS; GNRI was significantly correlated with age (*p* < 0.001), sex (*p* = 0.048), body mass index (BMI) (*p* < 0.001), albumin (*p* < 0.001), pulmonary disease (*p* = 0.026), ASA-PS score (*p* = 0.002), depth of tumor invasion (*p* < 0.001), lymph node metastasis (*p* < 0.001), lymphatic invasion (*p* < 0.001), and pathological stage (*p* < 0.001). Table 3 summarizes the relationship between the GNRI and operation-related factors based on OS, the GNRI was significantly correlated with operation duration (*p* < 0.001), bleeding amount (*p* = 0.009), type of procedure (*p* = 0.001), type of approach (*p* < 0.001), death due to primary disease (*p* = 0.001), and death due to other disease (*p* < 0.001) (Table 3).

**Table 1:** Receiver operating characteristic curve analysis for OS and DSS

Analysis for OS			Analysis for DSS		
Variable	AUC	*p* value	Variable	AUC	*p* value
GNRI	0.637	< 0.001	GNRI	0.645	< 0.001
Albumin	0.635	< 0.001	Albumin	0.639	< 0.001
PNI	0.631	< 0.001	CA19–9	0.634	0.002
CA19–9	0.590	0.002	PNI	0.623	0.002
CEA	0.577	0.006	CEA	0.591	0.028
mGPS	0.573	0.007	mGPS	0.574	0.068
CAR	0.572	0.009	CAR	0.557	0.155
CRP	0.56	0.028	CRP	0.543	0.285
NLR	0.542	0.126	NLR	0.541	0.311
PLR	0.475	0.363	PLR	0.524	0.550

*CAR* C-reactive protein/albumin ratio, *CA19–9* carbohydrate antigen 19–9, *CEA* carcinoembryonic antigen, *CRP* C-reactive protein, *DSS* disease-specific survival, *GNRI* geriatric nutritional risk index, *mGPS* modified Glasgow prognostic score, *NLR*, *OS* overall survival; neutrophil/lymphocyte ratio, *PLR* platelet/lymphocyte ratio, *PNI* prognostic nutritional index

**Table 2:** Relationship of GNRI with patient characteristics based on OS and DSS

		Based on OS (cutoff, 97.0)			Based on DSS (cutoff, 95.8)		
	All (*n* = 497)	GNRI^High^ (*n* = 269)	GNRI^Low^ (*n* = 228)	*p* value	GNRI^High^ (*n* = 289)	GNRI^Low^ (*n* = 208)	*p* value
Age (years)	80.6 ± 4.0	79.7 ± 3.6	81.7 ± 4.2	< 0.001	79.8 ± 3.6	81.7 ± 4.2	< 0.001
Sex				0.048			0.071
Male	325 (65.4)	189 (70.3)	141 (61.8)		196 (67.8)	129 (62.0)	
Female	164 (34.6)	80 (29.7)	87 (38.2)		85 (32.2)	79 (48.0)	
BMI	22.2 ± 3.3	23.2 ± 2.7	21.0 ± 3.5	< 0.001	23.1 ± 2.7	20.9 ± 3.5	< 0.001
Albumin	3.82 ± 0.54	4.18 ± 0.25	3.38 ± 0.47	< 0.001	4.16 ± 0.26	3.34 ± 0.47	< 0.001
Comorbidity							
Cardiac disease	15 (3.0)	8 (3.0)	7 (3.1)	0.950	10 (3.5)	5 (2.4)	0.497
Pulmonary disease	27 (5.4)	9 (3.3)	18 (7.9)	0.026	12 (4.2)	15 (7.2)	0.138
Diabetes mellitus	89 (17.9)	56 (20.8)	33 (14.5)	0.066	58 (20.1)	31 (14.9)	0.138
ASA-PS score				0.002			0.002
1	42 (8.4)	23 (8.6)	19 (8.3)		25 (8.7)	17 (8.2)	
2	366 (73.6)	213 (79.2)	153 (67.1)		227 (78.5)	139 (66.8)	
3	89 (18.0)	33 (12.2)	56 (24.6)		37 (12.8)	52 (25.0)	
Depth of tumor invasion				< 0.001			< 0.001
T1	268 (53.9)	177 (65.8)	91 (39.9)		189 (65.4)	79 (38.0)	
T2	73 (14.7)	31 (11.5)	42 (18.4)		32 (11.1)	41 (19.7)	
T3	104 (20.9)	40 (14.9)	64 (28.1)		45 (15.6)	59 (28.4)	
T4	52 (10.5)	21 (7.8)	31 (13.6)		23 (7.9)	29 (13.9)	
Lymph node metastasis				< 0.001			< 0.001
Present	161 (32.4)	68 (25.3)	93 (40.8)		75 (26.0)	86 (41.3)	
Absent	336 (67.6)	201 (74.7)	135 (59.2)		214 (74.0)	122 (58.7)	
Histology				0.670			0.464
Differentiated	334 (67.2)	183 (68.0)	151 (66.2)		198 (68.5)	136 (65.4)	
Undifferentiated	163 (32.8)	86 (32.0)	77 (33.8)		91 (31.5)	72 (34.6)	
Lymphatic invasion				0.012			0.004
Present	295 (59.4)	146 (54.3)	149 (65.4)		156 (54.0)	139 (66.8)	
Absent	202 (40.6)	123 (45.7)	79 (34.6)		133 (46.0)	69 (33.2)	
Venous invasion				0.115			0.062
Present	236 (47.5)	119 (44.2)	117 (51.3)		127 (43.9)	109 (52.4)	
Absent	261 (52.5)	150 (55.8)	111 (48.7)		162 (56.1)	99 (47.6)	
pStage				< 0.001			< 0.001
I	296 (59.6)	190 (70.6)	106 (46.5)		202 (69.9)	94 (45.2)	
II	123 (24.7)	47 (17.5)	76 (33.3)		53 (18.3)	70 (33.7)	
III	78 (15.7)	32 (11.9)	46 (20.2)		34 (11.8)	44 (21.1)	

Data are presented as means ± standard deviation or numbers (percentages) of patients

*ASA-PS* American Society of Anesthesiologists physical status, *BMI* body mass index, *DSS* disease-specific survival, *GNRI* geriatric nutritional risk index, *OS* overall survival, *pStage* pathological stage

**Table 3:** Relationship of GNRI with operation-related factors based on OS and DSS

		Based on OS (cutoff, 97.0)			Based on DSS (cutoff, 95.8)		
	All (*n* = 497)	GNRI^High^ (*n* = 269)	GNRI^Low^ (*n* = 228)	*p* value	GNRI^High^ (*n* = 289)	GNRI^Low^ (*n* = 208)	*p* value
Operation duration (min)	244 ± 142	262 ± 151	224 ± 129	< 0.001	264 ± 149	219 ± 126	< 0.001
Bleeding amount (mL)	279 ± 402	240 ± 325	326 ± 474	0.009	241 ± 335	329 ± 469	0.002
Procedure				0.001			0.002
Distal	325 (65.4)	186 (69.1)	139 (61.0)		200 (69.2)	125 (60.1)	
Proximal	34 (6.8)	25 (9.3)	9 (3.9)		25 (8.7)	9 (4.3)	
Total	138 (27.8)	58 (21.6)	80 (35.1)		64 (22.1)	74 (35.6)	
Approach				< 0.001			< 0.001
Laparoscopic	217 (43.7)	149 (55.4)	68 (29.8)		163 (56.4)	54 (26.0)	
Open	280 (56.3)	120 (44.6)	160 (70.2)		126 (43.6)	154 (74.0)	
Adjuvant chemotherapy				0.372			0.529
Present	73 (14.7)	36 (13.4)	37 (16.2)		40 (13.8)	33 (15.9)	
Absent	424 (85.3)	233 (86.6)	191 (83.8)		249 (86.2)	175 (84.1)	
Death due to primary disease				0.001			< 0.001
Present	58 (11.7)	20 (7.4)	38 (16.7)		21 (7.3)	37 (17.8)	
Absent	439 (88.3)	249 (92.6)	190 (83.3)		268 (92.7)	171 (82.2)	
Death due to other diseases				< 0.001			0.011
Present	113 (22.7)	45 (16.7)	68 (29.8)		54 (18.7)	59 (28.4)	
Absent	384 (77.3)	224 (83.3)	160 (70.2)		235 (81.3)	149 (71.6)	

Data are presented as means ± standard deviation or numbers (percentages) of patients

*DSS* disease-specific survival, *GNRI* geriatric nutritional risk index, *OS* overall survival, *pStage* pathological stage

**Fig. 3:**
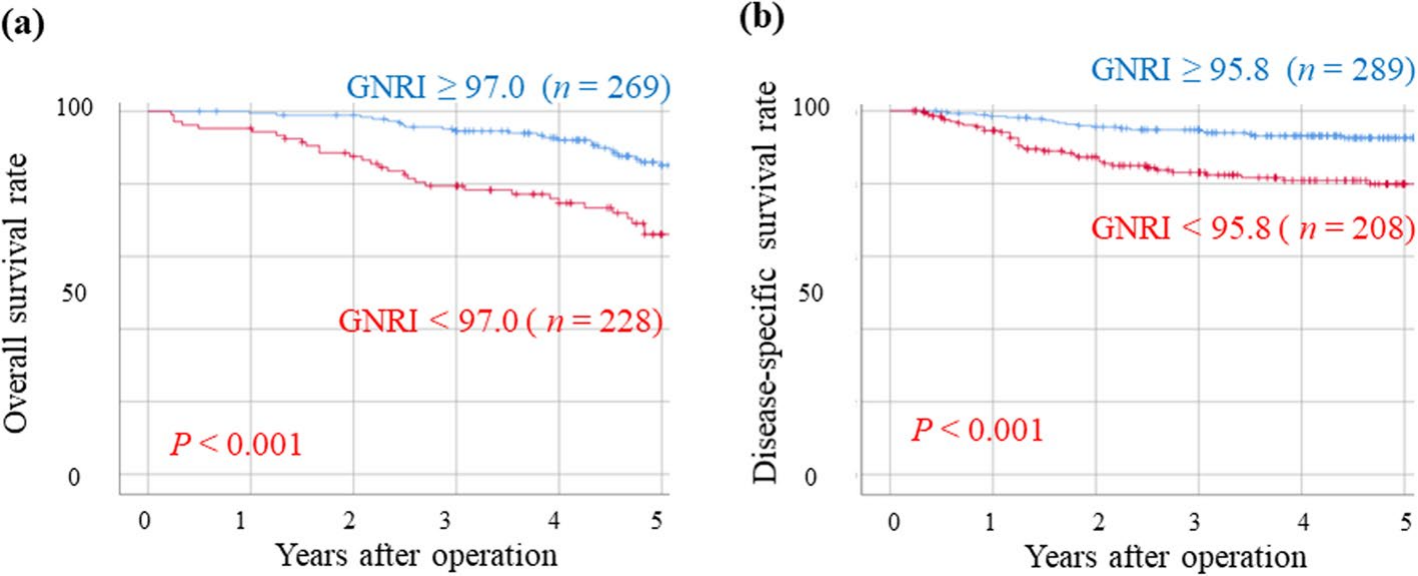
Overall survival (**a**) and disease-specific survival (**b**) curves in elderly patients with gastric cancer. Abbreviations: GNRI, geriatric nutritional risk index

The OS rates were significantly worse in the GNRI^Low^ group than in the GNRI^High^ group (*p* < 0.001) (Fig. 3a). The univariate analysis revealed that the OS was significantly worse in patients aged ≥80 years, and those with undifferentiated adenocarcinoma, positive lymphatic invasion, positive venous invasion, T2 or deeper tumor invasion, positive lymph node metastasis, positive venous invasion, bleeding ≥206 mL, and low GNRI (Table 4). By multivariate analysis, low GNRI, age ≥ 80 years, positive lymph node metastasis, and bleeding ≥206 mL were independent prognostic factors for OS (Table 4).

**Table 4:** Univariate and multivariate analysis in elderly patients with gastric cancer for OS

Variable	Univariate analysis			Multivariate analysis		
	Hazard ratio	95% CI	*p* value	Hazard ratio	95% CI	*p* value
Age (≥80 vs < 80 years)	2.132	1.542–2.947	< 0.001	1.673	1.196–2.340	0.003
Sex (female vs male)	0.944	0.682–1.307	0.728			
BMI (< 22.0 vs ≥22.0 kg/m^2^)	1.211	0.895–1.637	0.214			
ASA-PS score (2.3 vs 1)	1.843	0.993–3.423	0.053			
Histology (undifferentiated vs differentiated)	1.606	1.183–2.180	0.002	1.362	0.996–1.864	0.053
Lymphatic invasion (present vs absent)	2.326	1.657–3.266	< 0.001	0.900	0.552–1.466	0.671
Venous invasion (present vs absent)	2.007	1.475–2.732	< 0.001	1.364	0.928–2.005	0.114
pT (≥2 vs 1)	2.698	1.973–3.691	< 0.001	1.278	0.867–1.883	0.215
pN (present vs absent)	3.101	2.287–4.205	< 0.001	2.123	1.465–3.079	< 0.001
Operation duration (≥154 vs < 154 min)	1.054	0.571–1.946	0.866			
Bleeding (≥206 vs < 206 mL)	1.903	1.407–2.572	< 0.001	1.572	1.151–2.147	0.008
GNRI (< 97.0 vs ≥97.0)	2.454	1.799–3.348	< 0.001	1.905	1.374–2.641	< 0.001

*ASA-PS* American Society of Anesthesiologists physical status, *BMI* body mass index, *CI* confidence interval, *GNRI* geriatric nutritional risk index, *OS* overall survival, *pT* pathological depth of invasion, *pN* pathological lymph node metastasis

### The utility of GNRI according to DSS

Table 1 shows the AUC for each potential prognostic factor based on the ROC curve analysis for DSS. Among these, the GNRI had the highest AUC and the highest predictive value. Using the optimal GNRI cutoff value of 95.8 determined with the ROC analysis (Fig. 2b), the patients were divided into the high GNRI (GNRI^High^, *n* = 289) and low GNRI (GNRI^Low^, *n* = 208) groups. As shown in Table 2, the GNRI was significantly correlated with the following clinicopathological factors: age (*p* < 0.001), BMI (*p* < 0.001), albumin (*p* < 0.001), ASA-PS score (*p* = 0.002), depth of tumor invasion (*p* < 0.001), lymph node metastasis (*p* < 0.001), lymphatic invasion (*p* = 0.004), and pathological stage (*p* < 0.001). In addition, the GNRI was significantly correlated with the following operative factors: operation duration (*p* < 0.001), bleeding amount (*p* = 0.002), type of procedure (*p* = 0.002), type of approach (*p* < 0.001), death due to primary disease (*p* < 0.001), and death due to other disease (*p* = 0.011) (Table 3).

The DSS rates were significantly worse in the GNRI^Low^ group than in the GNRI^High^ group (*p* < 0.001) (Fig. 3b). The univariate analysis indicated that the DSS was significantly worse in patients with undifferentiated adenocarcinoma, higher tumor size, positive lymphatic invasion, positive venous invasion, T2 or deeper tumor invasion, positive lymph node metastasis, positive venous invasion, bleeding ≥206 mL, and low GNRI (Table 5). The multivariate analysis indicated that low GNRI, undifferentiated adenocarcinoma, positive venous invasion, T2 or deeper tumor invasion, positive lymph node metastasis, and bleeding ≥206 mL were independent prognostic factors for DSS (Table 5).

**Table 5:** Univariate and multivariate analysis in elderly patients with gastric cancer for DSS

Variable	Univariate analysis			Multivariate analysis		
	Hazard ratio	95% CI	*p* value	Hazard ratio	95% CI	*P* value
Age (≥80 vs < 80 years)	1.548	0.909–2.634	0.107			
Sex (female vs male)	1.185	0.694–2.025	0.534			
BMI (< 22.0 vs ≥22.0 kg/m^2^)	1.029	0.614–1.722	0.914			
ASA-PS score (2.3 vs 1)	1.097	0.438–2.753	0.843			
Histology (undifferentiated vs differentiated)	2.582	1.541–4.326	< 0.001	1.881	1.114–3.175	0.018
Lymphatic invasion (present vs absent)	15.156	4.739–48.463	0.001	1.370	0.371–5.062	0.637
Venous invasion (present vs absent)	5.477	2.839–10.566	< 0.001	2.263	1.126–4.545	0.022
pT (≥2 vs 1)	41.223	10.056–168.996	< 0.001	11.642	2.690–50.397	< 0.001
pN (present vs absent)	10.522	5.565–19.895	< 0.001	3.621	1.849–7.091	< 0.001
Operation duration (≥154 vs < 154 min)	1.301	0.407–4.161	0.657			
Bleeding (≥206 vs < 206 mL)	3.230	1.879–5.551	< 0.001	2.137	1.236–3.695	0.007
GNRI (< 95.8 vs ≥95.8)	3.059	1.778–5.263	< 0.001	1.780	1.025–3.025	0.043

*ASA-PS* American Society of Anesthesiologists physical status, *BMI* body mass index, *CI* confidence interval, *DSS* disease-specific survival, *GNRI* geriatric nutritional risk index, *pT* pathological depth of invasion, *pN* pathological lymph node metastasis

## Discussion

In the present retrospective multicenter study, the GNRI emerged as a prognostic factor for OS and DSS with the best predictive performance among several inflammation and nutritional markers. Although there have been reports to identify the utility of GNRI in elderly gastric cancer patients aged over 65 years, no studies currently address patients over 75 years. To the best of our knowledge, this is the first multicenter study report to clarify the usefulness of GNRI as a prognostic marker in this patient population. Prediction of prognosis in elderly patients is fraught with issues due to the increased frequency of comorbidities and death due to other causes unique to the elderly. Therefore, the GNRI is a clinically useful tool that may be considered for prognostic prediction in conjunction with the TNM classification and other measures of disease progression.

The utility of inflammation and nutritional markers, such as CRP-based CAR and platelet-based PLR related to inflammation, lymphocyte-based NLR related to immunity, and albumin-based PNI related to nutrition, has been extensively investigated in patients with gastric cancer; however, few reports focused on the elderly patients with gastric cancer [14, 23, 24, 26]. In the present multicenter study with a relatively large number of elderly patients with gastric cancer, the AUC values of albumin-based markers related to nutrition, such as the GNRI, PNI, and the modified Glasgow prognostic score, were higher for both OS and DSS, indicating the importance of nutritional status on these outcomes. Malnutrition can be caused by physical, psychological, or physiological changes associated with aging, leading to decreased resistance to infection, immune function, and quality of life [27, 28]. In addition, gastric cancer can easily lead to malnutrition due to impaired food passage. CAR, NLR, and PNI are well-known prognostic factors for gastric cancer; however, the AUC value was higher for the GNRI than CAR, NLR, and PNI in the present study, indicating that CRP, which is related to inflammation, and lymphocyte count, which is related to immunity, might have less utility in predicting the prognosis of elderly patients with gastric cancer compared to the GNRI, which includes strong nutritional components. The GNRI was developed as an objective and simple screening tool to assess nutrition-related risk of morbidity and mortality in elderly hospitalized patients. The GNRI can be easily calculated using albumin, body weight, and height. Albumin, a major protein in human serum, reflects the nutritional status of an individual, and hypoalbuminemia has been demonstrated to be associated with poor prognosis in patients with various cancer [29–31]. Body weight has been reported as an indicator of both systemic disease severity and protein and calorie stores; its association with prognosis in patients with cancer has also been reported [32–34]. BMI, which takes weight and height into account, is another commonly used parameter to assess the nutritional status of individuals [35, 36]. The GNRI can be easily calculated using albumin and BMI and may be useful as a prognostic indicator for gastric cancer in the elderly.

Our main finding of the GNRI as a prognostic factor in patients with gastric cancer is in agreement with the outcomes of a study by Hirahara et al., who retrospectively examined 297 elderly patients over 65 years of age who underwent curative laparoscopic gastrectomy for gastric cancer. While the age cutoff was 65 years in that study, similar to our findings, the authors reported that the GNRI was significantly associated with OS and cancer-specific survival in elderly patients with gastric cancer and that the GNRI was an independent predictor of OS [37]. Similarly, Sugawara et al. reported that the GNRI was a prognostic factor in gastric cancer based on its significant association with OS and cancer-specific survival in a retrospective analysis of 1166 patients who underwent curative gastrectomy, although the study included patients across all ages and was not restricted to older patients [38]. Nevertheless, our study adds to the accumulating evidence that the GNRI might be considered as a prognostic factor in patients with gastric cancer while confirming its utility in elderly patients.

In this study, the prognosis was worse in the GNRI^Low^ group for both OS and DSS; however, the mechanism underlying this outcome is unclear. It is possible that the stage of cancer, one of the clinicopathological factors, might have been more advanced in patients with low GNRI. Previous studies reported the association of low GNRI with advanced stage in various cancers [19, 37, 39–41], similar to our findings. Various cytokines are released in advanced cancer [42–44], and some studies reported that increased inflammation markers and decreased nutritional marker were associated with increased catabolism, resulting in anorexia and a negative effect on nutritional status [45, 46], which may also account for a low GNRI.

In this study, the low GNRI was significantly correlated with deeper depth of tumor invasion, positive lymph node metastasis, and advanced pathological stage. Generally, advanced cancer tends to be treated with open surgery, which may explain the lower rate of patients undergoing laparoscopic surgery in the GNRI^Low^ group than in the GNRI^High^ group [47]. Laparoscopic surgery is reported to require long operative time and results in low blood loss [48], which may be the reason for the longer operation duration and lower blood loss in the GNRI^High^ group than in the GNRI^Low^ group.

This study has several limitations. First, this was a retrospective analysis; however, the study included multiple institutions. Second, the optimal GNRI cutoff value in elderly patient with gastric cancer is unknown. Third, the study did not include data on patients younger than 75 years of age. Fourth, the definition of elderly used in the present study was different from that used in some of the other studies. Currently, the Japanese Geriatrics Society has proposed to redefine the elderly as 75 years of age or older, and thus we adopted a threshold of 75 years [49].

## Conclusion

The GNRI exhibited the best prognostic performance among several inflammation and nutritional markers in elderly patients with gastric cancer undergoing curative gastrectomy. As a simple and cost-effective tool, the GNRI is a promising index for predicting OS and DSS in elderly patients with gastric cancer.

## Supplementary Information

**Additional file 1: Supplemental Table.** Names of the ethics committees of all participating institutions and the reference numbers of this study.

## Data Availability

The datasets used and analyzed in the present study are not publicly available due to the information that could compromise the privacy of research participants but are available from the corresponding author on reasonable request.

## Abbreviations

ASA-PS: American Society of Anesthesiologists physical status
BMI: Body mass index
CAR: C-reactive protein/albumin ratio
CEA: Carcinoembryonic antigen
CRP: C-reactive protein
CT: Computed tomography
GNRI: Geriatric nutritional risk index
NLR: Neutrophil/lymphocyte ratio
OS: Overall survival
PLR: Platelet/lymphocyte ratio
PNI: Prognostic nutritional index
