# Supplementary material for: Impact of geriatric nutritional risk index on outcomes after gastrectomy in elderly patients with gastric cancer: a retrospective multicenter study in Japan
Source: BMC Cancer. 2022 May 12;22:540. doi: 10.1186/s12885-022-09638-6 (PMC9103416; doi:10.1186/s12885-022-09638-6)
Supplement: Supplementary file 1 — Additional file 1: Supplemental Table. Names of the ethics committees of all participating institutions and the reference numbers of this study. [file 12885_2022_9638_MOESM1_ESM.docx]

**Supplemental Table.** Names of the ethics committees of all participating institutions and the reference numbers of this study

| Name of institution | Name of the ethics committee | Reference number |
| --- | --- | --- |
| Tottori University Hospital | Tottori university hospital ethics committee | 17A133 |
| Japanese Red Cross Tottori Hospital | Japanese red cross tottori hospital ethics committee | 69 |
| Tottori Prefectural Central Hospital | Tottori Prefectural Central Hospital ethics committee | 2017-67 |
| National Hospital Organization, Hamada Medical Center | Hamada medical center ethics committee | 3071 |
| Tottori Prefectural Kousei Hospital | Tottori prefectural kousei hospital ethics committee | 137 |
| Sanin Rosai Hospital | Sanin Rosai Hospital ethics committee | 2018-08 |
| Yonago Medical Center | Yonago Medical Center ethics committee | 3007-01 |
| Japanese Red Cross Masuda Hospital | Masuda red cross hospital medical ethics committee | 55 |
| Tottori Seikyo Hospital | Tottori Seikyo Hospital ethics committee | 2018023 |
| Yasugi municipal hospital | Yasugi municipal hospital ethics committee | H30-2 |
| Hakuai Hospital | Hakuai Hospital ethics committee | 20200004 |
| Saihaku Hospital | Saihaku Hospital ethics committee | 18081 |
